# The presence of contrast agent increases organ radiation dose in contrast-enhanced CT

**DOI:** 10.1007/s00330-021-07763-7

**Published:** 2021-03-30

**Authors:** Mahta Mazloumi, Gert Van Gompel, Veerle Kersemans, Johan de Mey, Nico Buls

**Affiliations:** 1 Department of Radiology, Vrije Universiteit Brussel (VUB), Universitair Ziekenhuis Brussel (UZ Brussel), Laarbeeklaan 101, 1090 Brussels, Belgium; 2 Department of Oncology, University of Oxford, Headington, Oxford, OX3 7LE UK

**Keywords:** Tomography, X-ray Computed, Iodine, Radiation dosage, Humans, Phantoms, Imaging

## Abstract

**Objectives:**

Routine dosimetry calculations do not account for the presence of iodine in organs and tissues during CT acquisition. This study aims to investigate the impact of contrast agent (CA) on radiation dose.

**Methods:**

First, relation between absorbed radiation dose and iodine concentrations was investigated using a cylindrical water phantom with iodine-saline dilution insertions. Subsequently, a retrospective study on abdominal dual-energy CT (DECT) patient data was performed to assess the increase of the local absorbed radiation dose compared to a non-contrast scan. Absorbed doses were estimated with Monte Carlo simulations using the individual CT voxel data of phantom and patients. Further, organ segmentations were performed to obtain the dose in liver, liver parenchyma, left kidney, right kidney, aorta, and spleen.

**Results:**

In the phantom study, a linear relation was observed between the radiation dose normalized by computed tomography dose index (CTDI) and CA concentrations I_conc_ (mg/ml) for three tube voltages; $\frac{D_{80 kVp}}{CTDI_{vol}}$ = 0.14 × I_conc_ + 1.02, $\frac{D_{120 kVp}}{CTDI_{vol}}$ = 0.16 × I_conc_ + 1.21, $\frac{D_{140 kVp}}{CTDI_{vol}}$ = 0.16 × I_conc_ + 1.24, and for DECT acquisition; $\frac{D_{DECT}}{CTDI_{vol}}$ = 0.15 × I_conc_ + 1.09. Similarly, a linear relation was observed between the dose increase and the organ iodine contents (*R*^2^ = 0.86 and *p*_value_ < 0.01) in the patient study. The relative doses increased in the liver (21 ± 5%), liver parenchyma (20 ± 5%), right kidney (37 ± 7%), left kidney (39 ± 7%), aorta (34 ± 6%) and spleen (26 ± 4%). In addition, the local dose distributions changed based on patient’s anatomy and physiology.

**Conclusions:**

Compared to a non-contrast scan, the organ doses increase by 30% in contrast-enhanced abdominal CT. This study suggests considering CA in dosimetry calculations, epidemiological studies, and organ dose estimations while developing new CT protocols.

**Key Points:**

*• The presence of contrast media increases radiation absorption in CT, and this increase is related to the iodine content in the organs.*

*• The increased radiation absorption due to contrast media can lead to an average 30% increase in absorbed organ dose.*

• *Iodine should be considered in CT radiation safety studies*.

## Introduction

The use of contrast agents (CAs) is essential in medical imaging to opacify lesions and tissues. In CT alone, 50–60% of all procedures use iodine CA to ensure diagnostic quality [1, 2]. A considerable amount of literature has been published on the safety of clinical CA’s, and it is well acknowledged that respecting guidelines provides its safe and effective use [3, 4]. In recent years, researchers have examined the impact of iodine CA on radiation-induced biological effects. Collectively, these studies outline a critical role for CA to increase the number of radiation-induced DNA double-strand breaks (DSBs) compared to unenhanced imaging [5–10]. The biological damage caused by CA is a physics phenomenon rather than chemical, as there is no evidence that the presence of CA leads to DNA damage in the absence of radiation [5]. This well-known phenomenon occurs as CA increases the attenuation [11, 12] due to photoelectric effect, which consequently increases the locally absorbed radiation dose.

The impact of CA’s on absorbed radiation dose in organs and tissues has recently been challenged by a few studies, which demonstrate a dose increase in the presence of iodine [2, 13–15]. However, research on the subject has been mostly restricted to simplified geometrical models, homogenous organ structures, and single CT acquisitions. To date, routine CT dosimetry approaches such as dose length product (DLP) to effective dose (E) conversion coefficients that are determined by organs’ anatomical locations (so-called k-factors) [16], experimental humanoid phantoms, and commercial CT dosimetry such as CT-Expo [17], NCICT [18], and imPACT CT patient dosimetry calculator (imPACT) do not account for the presence of CA.

Including iodine in radiation dose calculation needs advanced Monte Carlo (MC) simulations. We used ImpactMC (Advanced Breast-CT) MC software which is specifically designed and validated for CT dosimetry [19] and has been used in various clinical studies [20–24]. We chose to use ImpactMC over the existing MC codes such as Penelope [25], Geant4 [26], MCNP [27], and EGS4 [28] because it allows using the actual clinical data as a geometrical model, and it provides a flexible environment to include parameters such as table increment, helical scan mode, and tube current modulation.

This study attempts to provide a more accurate organ dose estimation of contrast-enhanced CT. First, we investigated the relationship between absorbed radiation dose and iodine contrast concentration in a phantom study. Secondly, we performed a patient study to address the local radiation dose in organs and tissues including the liver, liver parenchyma, right kidney, left kidney, aorta, and spleen in the hepatic phase of contrast-enhanced abdominal CT. The study was carried out by performing ad hoc Monte Carlo dosimetry simulations which use patient-specific CT images of each individual as a geometrical model and estimates the iodine content of each organ, based on information obtained from dual-energy computed tomography (DECT) scans.

## Materials and methods

### Monte Carlo simulation model

MC simulations were performed using the MC software ImpactMC. The DICOM images obtained from CT and DECT acquisitions (Revolution CT, GE Healthcare) were used as 3D geometrical models in the phantom and patient study, respectively. The acquisitions were modeled based on ad hoc scan parameters including tube current, table height, beam collimation, number of rotations, rotation direction, rotation time, scan mode, bow-tie filter, X-ray spectrum, and the distance of plane in focus from the center of rotation and fan angle. The details of the four latter parameters were supplied by the CT manufacturer and are confidential. The details of the other parameters were obtained from the DICOM headers. A total number of 3.1 × 10^9^ photons, break energy of 10 keV, number of interactions of 10, and 36 projections per rotation were used in the simulations. A validation experiment with a computed tomography dose index (CTDI) dosimetry phantom showed that the average error between measured and simulated CTDI_vol_ in the MC software for 80 kVp, 120 kVp, and 140 kVp and 80-mm collimation were 2.7%, 0.9%, and 0.7%, respectively.

### Phantom study

The goal of this part of the study was to investigate the relationship between radiation dose and iodine concentration. Eleven syringes were filled with diluted iodine solutions (mg I/ml) of 0.00 ± 0.00, 2.00 ± 0.06, 4.00 ± 0.12, 6.00 ± 0.18, 8.00 ± 0.24, 10.00 ± 0.30, 12.00 ± 0.37, 14.00 ± 0.43, 16.00 ± 0.50, 18.00 ± 0.57, and 20.00 ± 0.64. A 370 mg I/ml contrast media (Iopromide, Bayer healthcare) and saline (NaCl 0.9%) were used for formulating the dilutions. Each syringe (diameter = 2 cm) was placed in the middle of a water phantom (diameter = 21 cm) to represent a large blood vessel, such as the abdominal aorta which has a reported diameter ranging from 1.86 to 2.13 cm [29]. The phantom was scanned with similar acquisition parameters (helical, 80-mm collimation, large bow-tie filter). We performed the phantom study for tube voltages of 80 kVp, 120 kVp, and 140 kVp to represent a range between low and high tube voltages typically used in the clinic. For each scan, the tube current-time product (mAs) was adjusted to obtain a similar dose level (CTDI_vol_ = 9.91 ± 0.02 mGy). In addition, a DECT (80–140 kVp) acquisition was performed with the similar acquisition parameters, and the tube current-time product was adjusted (0.5 s rotation time, 405 mA) to obtain a similar CTDI_vol_ value (9.91 mGy) to the previous scans. The images were reconstructed using standard convolutional kernel and ASiR-V 50% reconstruction algorithm, which was also used in the clinical image reconstructions. For each simulation, we used the images obtained from scanning the phantom with the specific tube voltage settings, considering the effect of kilovoltage peak (kVp) on HU values. For DECT, simulations were performed for 80-kVp and 140-kVp tube voltages using DECT scan data. Materials were defined in the simulations as water, air, and the eleven iodine solutions.

**Fig. 1:**
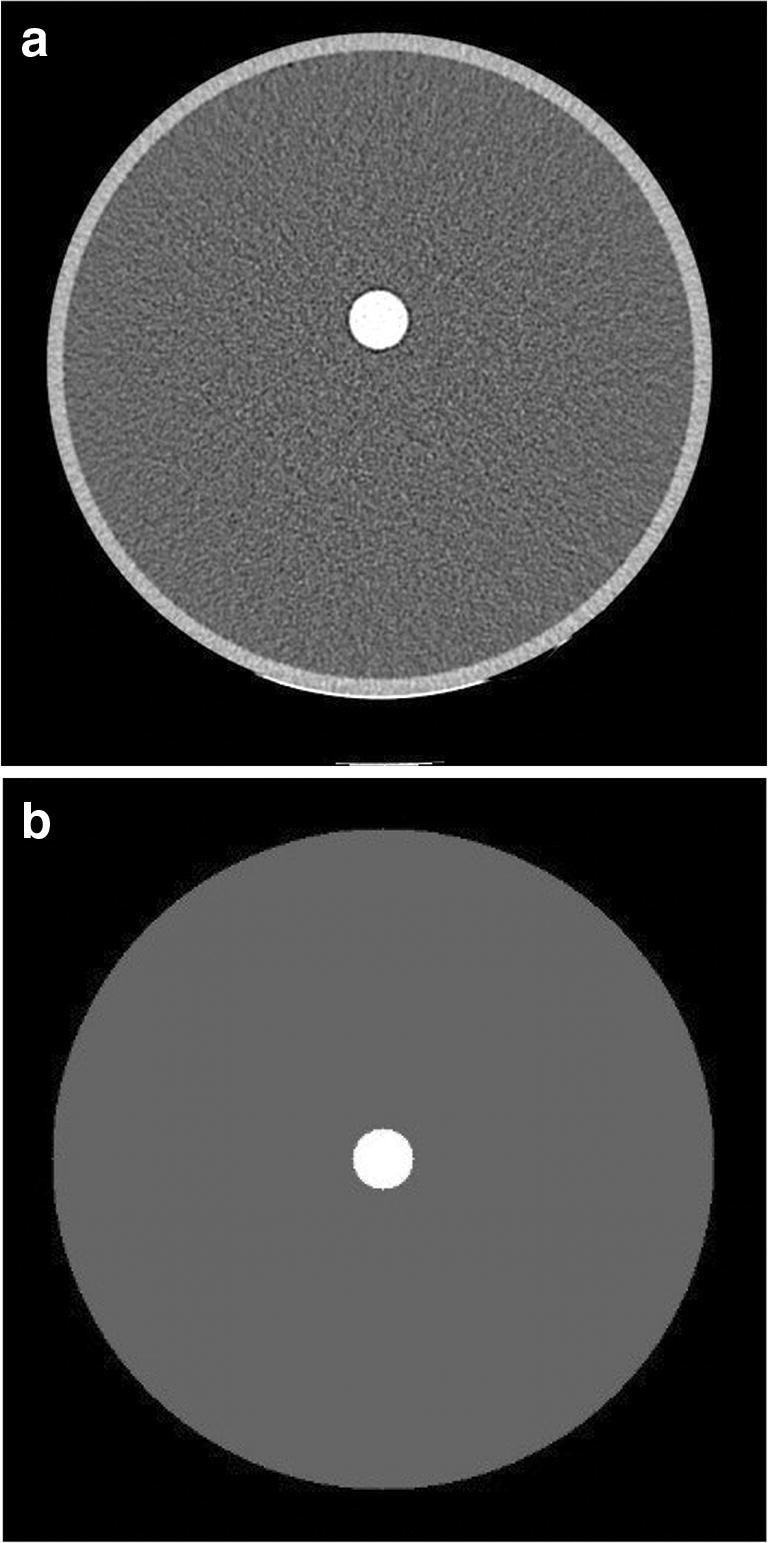
**a** CT image of the experimental phantom, and (**b**) the reconstructed virtual phantom

In addition, a virtual phantom model (Fig. 1b) with a similar dimension as the real phantom and without any HU noise (SD = 0) was considered to understand the net physics effect and to validate the experimental model (Fig. 1a). The virtual phantom consists of water and iodine dilutions. The polymethyl methacrylate (PMMA) was excluded in the virtual model to avoid beam hardening which is unavoidable in the real phantom model. We used MATLAB (Ver. R2020a) to build the virtual phantom as a DICOM volume where the HU for air and water were −1000 and 0, respectively. The HU for the iodine solutions were the average of HU in a ROI (diameter = 15 mm, depth = 25.6 mm) in the respecting iodine tube of the experimental phantom.

### Patient study

This study was approved by the institutional ethical committee (BUN 143201940531). DECT scans were considered in this study, because the material decomposition in DECT enables quantification of iodine distribution in tissues with an accuracy of ± 10% [30], as well as allowing to obtain virtual-unenhanced (VUE) images which resemble the same scan in the absence of contrast. We selected twenty abdominal DECT scans (rapid kV switching, GE Revolution CT), including ten males and ten females with a mean age of 53.1 years (min = 23 years, max = 92 years) from the hospital’s picture archiving and communication system (PACS), excluding patients with metallic implants or pacemakers. These scans were performed as a part of the patients’ routine diagnostic procedure to examine the gastrointestinal or urinary tract. All these scans were performed with DECT (80–140 kVp), large bow-tie filter, constant tube current, 80-mm collimation, and a helical acquisition. The scan parameters such as tube current, table increment, number of rotations, and scan time were varying between the patients. The patients were administered iodinated contrast: on average, 40.61 g (min = 30.4 g, max = 44.4 g) at a rate of 2.5 ml/s and a concentration of 320 (Iobitridol, Guerbet), 350 (Iomeprol, Bracco), or 370 (Iopromide, Bayer healthcare) mg I/ml. The presence of three different CAs in the patient study is due to changing the brand of CA on a rotational basis according to the hospital management. The images were reconstructed using standard convolutional kernel and ASiR-V 50% reconstruction algorithm. The virtual-unenhanced and contrast DECT images were selected to represent HU values in the absence and presence of contrast agent. Both series were processed at a monochromatic energy level of 68 keV to represent the effective energy of a 120-kVp scan. The iodine quantification (iodine map) of the patients was used to obtain the iodine content in the organs.

MC simulations were performed for 80-kVp and 140-kVp tube voltages using DECT scan data. The relation between HU and material density was defined by relating the HU of the voxels to their DECT iodine quantification densities. Materials were defined as iodine dilutions with varying concentrations of 0 to 20 mg I/ml incremented with steps of 0.1905 mg I/ml. After performing the simulations, 3D dose volumes in the presence (V_I_) and absence (V_I0_) of contrast were obtained which were used for data analysis.

### Data analysis and statistics

In the phantom study, mean dose values in voxels of a volumetric ROI with a diameter of 15 mm and depth of 25.6 mm were calculated for 80-kVp, 120-kVp, and 140-kVp tube voltages. The mean dose values of DECT scans were obtained with the similar approach, considering 2/3 ratio for the contribution of 80 kVp and 1/3 ratio for the contribution of 140 kVp in the radiation dose. The dose values were normalized by the CTDI_vol_ of the scans. The relationship between the CTDI_vol_ normalized dose and iodine was assessed by linear regression. In addition, the mean CTDI_vol_ normalized dose values in the virtual model and experimental model were compared using a Mann-Whitney *U* test.

In the patient study, six organs and tissues including the liver, liver parenchyma (liver excluding large vessels), right kidney, left kidney, aorta, and spleen were segmented on anatomical contrast CT images of the patients (open-source software 3D slicer [31]). As the obtained parametric dose maps overlaid the anatomical patient images, 3D organ dose volumes in the presence and absence of contrast were obtained by applying the segmented organ volumes as a mask on V_I_ and V_I0_, respectively. In addition, 3D HU volumes in the organs in the presence and absence of contrast, and 3D iodine volumes in the organs, were obtained by applying the segmented organ volumes as a mask on the contrast CT images, the VUE images, and the iodine quantification volume, respectively. The dose was reported as the mean value of all the voxels in the 3D organ dose volumes, considering 2/3 ratio for the contribution of 80 kVp and 1/3 ratio for the contribution of 140 kVp in the radiation dose. The mean HU value and the mean iodine value in each organ were reported as the mean value of all the voxels in the respecting 3D organ volume. The mean dose values were normalized by the size-specific dose estimates (SSDE, D_SSDE_) in order to make the results scanner and patient independent. The SSDE values were calculated based on AAPM report 2011 [32] using the CTDI_vol_ of the scans and the effective diameter of the patients. The relationship between %D_SSDE_ increase and iodine concentrations in the segmented organs was investigated by performing a Pearson correlation test. In addition, the average of absolute organ doses (excluding aorta) in the presence of CA $\left({\overline{\mathrm{D}}}_{\mathrm{I}}\right)$and absence of CA $\left({\overline{\mathrm{D}}}_0\right)$and their ratios defined as dose enhancement factor (DEF) were calculated for each patient.

## Results

### Results of the phantom study

**Fig. 2:**
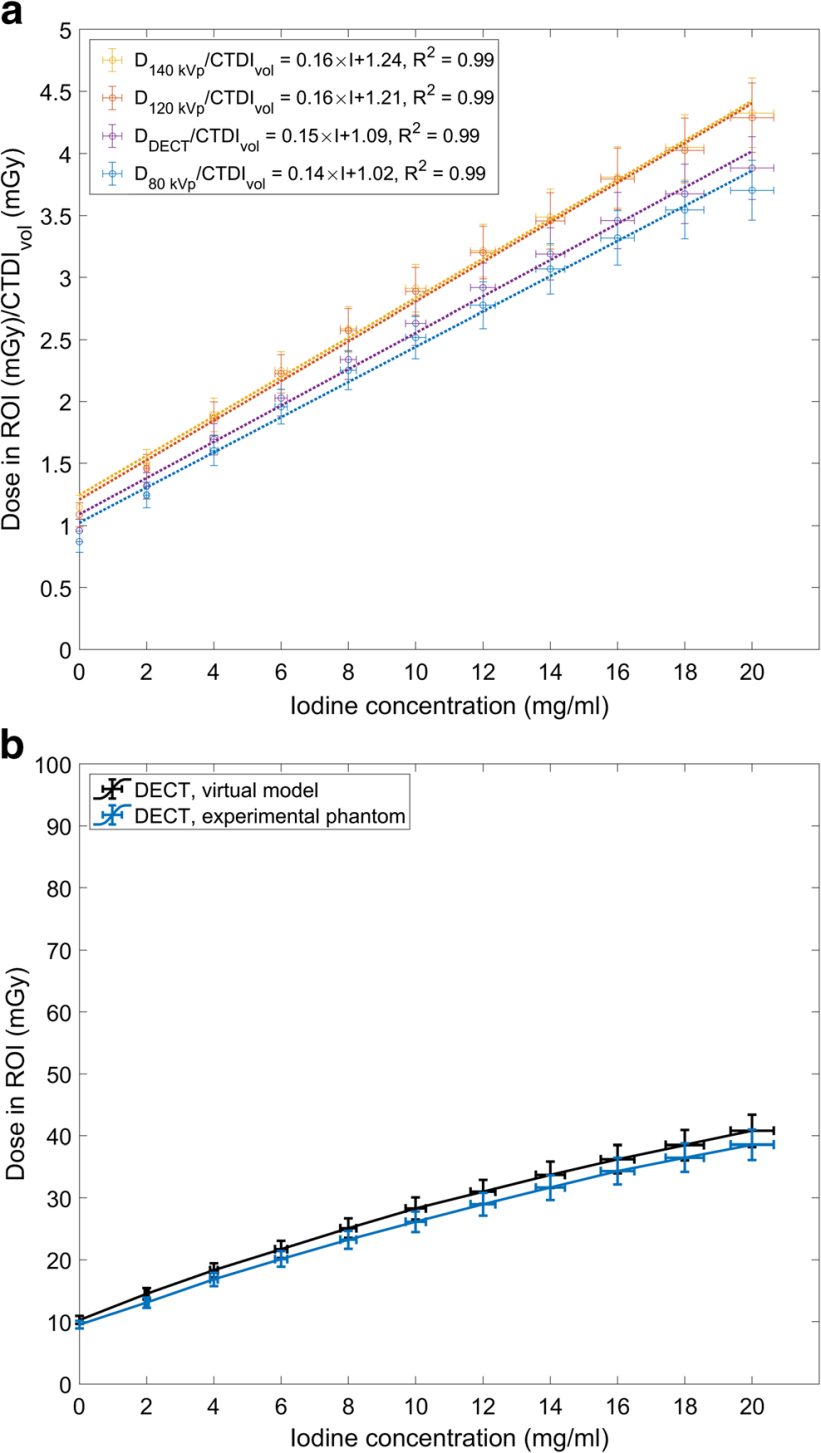
**a** CTDI_vol_ normalized dose versus iodine concentration in the ROI in syringes for 80, 120, 140 kVp, and DECT. **b** Dose in the ROI in the iodine syringes versus iodine concentration for virtual model and experimental phantom for DECT. Horizontal bars indicate uncertainty in the solutions and vertical bars indicate the uncertainty in the simulated dose (6.3%)

Results show a linear relation between the CTDI_vol_ normalized dose and the clinical range of CA concentrations I_conc_ (mg I/ml) (Fig. 2a). These equations are: $\frac{D_{80 kVp}}{CTDI_{vol}}$ = 0.14 × I_conc_ + 1.02$\left({R}^2=0.99\right),\frac{D_{120 kVp}}{CTDI_{vol}}$= 0.16 × I_conc_ + 1.21 (*R*^2^ = 0.99), $\frac{D_{140 kVp}}{CTDI_{vol}}$ = 0.16 × I_conc_ + 1.24 (*R*^2^ = 0.99), and $\frac{D_{DECT}}{CTDI_{vol}}$ = 0.15 × I_conc_ + 1.09 (*R*^2^ = 0.99). The dose results obtained from simulations of the virtual phantom were in good agreement with the dose results obtained from the experimental phantom with the mean ± SD difference of 6.9 ± 1.4% (min = 5.2%–max = 10%) (Fig. 2b, Mann-Whitney *U* test *p*_value_ = 0.74, ∝ = 0.05). The slightly higher dose values in the virtual phantom can be contributed to the absence of PMMA and as a result the absence of beam hardening due to PMMA.

### Results of the patient study

**Fig. 3:**
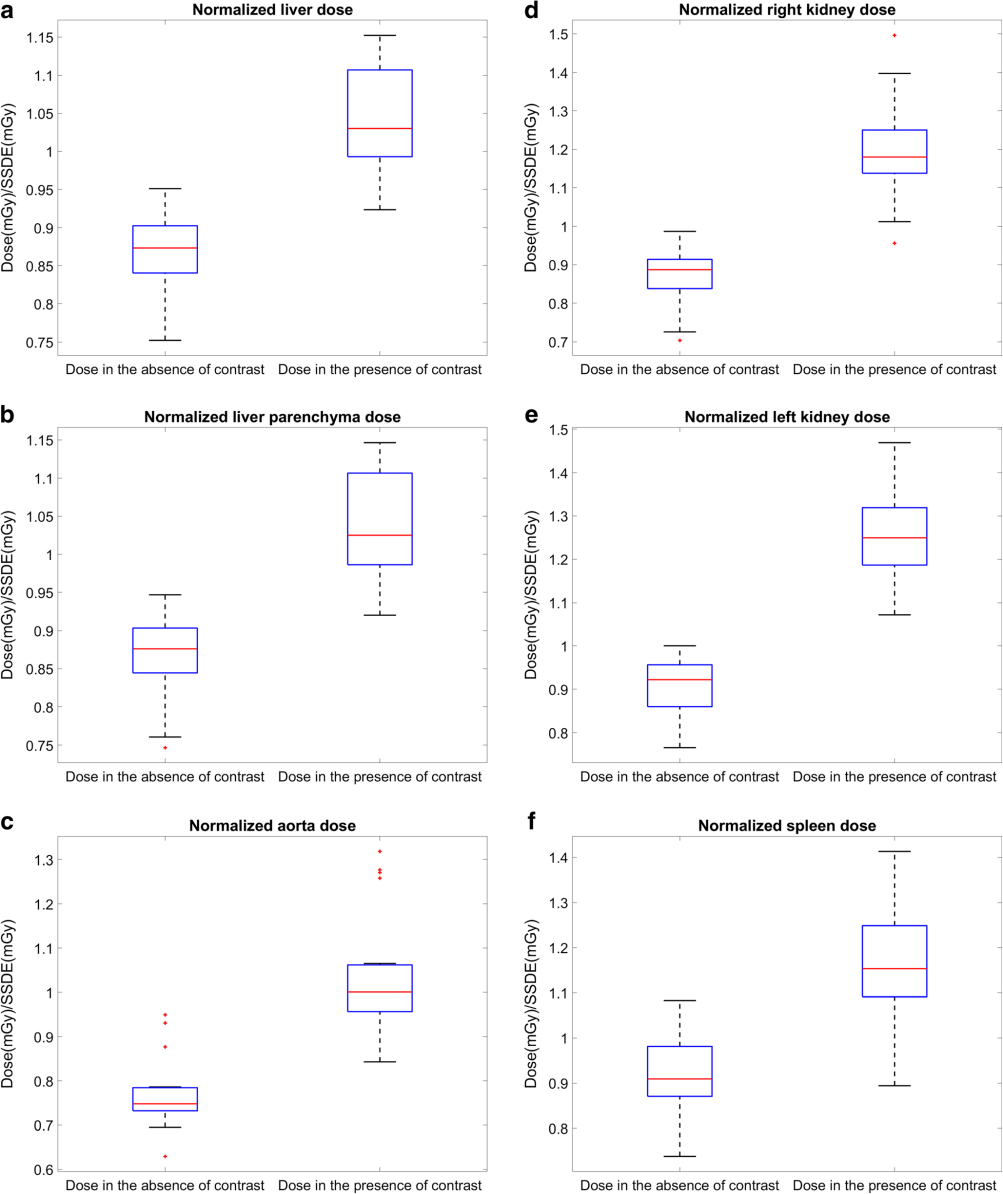
D_SSDE_ in the presence and in the absence of CA in (**a**) liver, (**b**) liver parenchyma, (**c**) aorta, (**d**) right kidney, (**e**) left kidney, and (**f**) spleen in the 20 patients

**Fig. 4:**
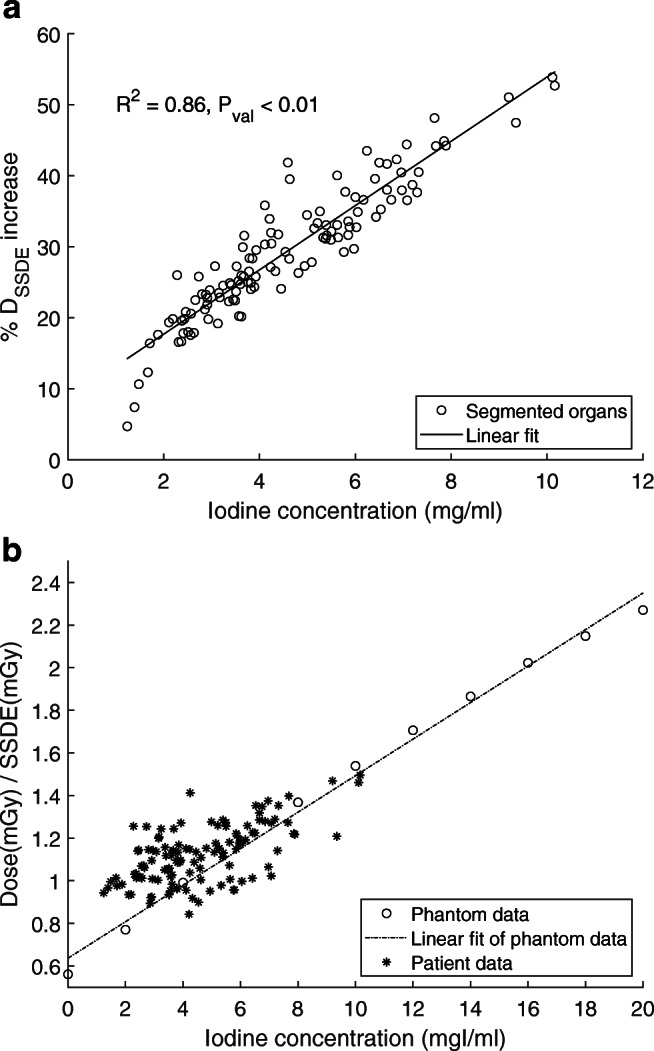
**a** A Pearson correlation test shows a linear agreement between %D_SSDE_ increase and iodine content in all the organs of the patient study. **b** D_SSDE_ in the segmented organs and iodine solutions with respect to their iodine content

**Fig. 5:**
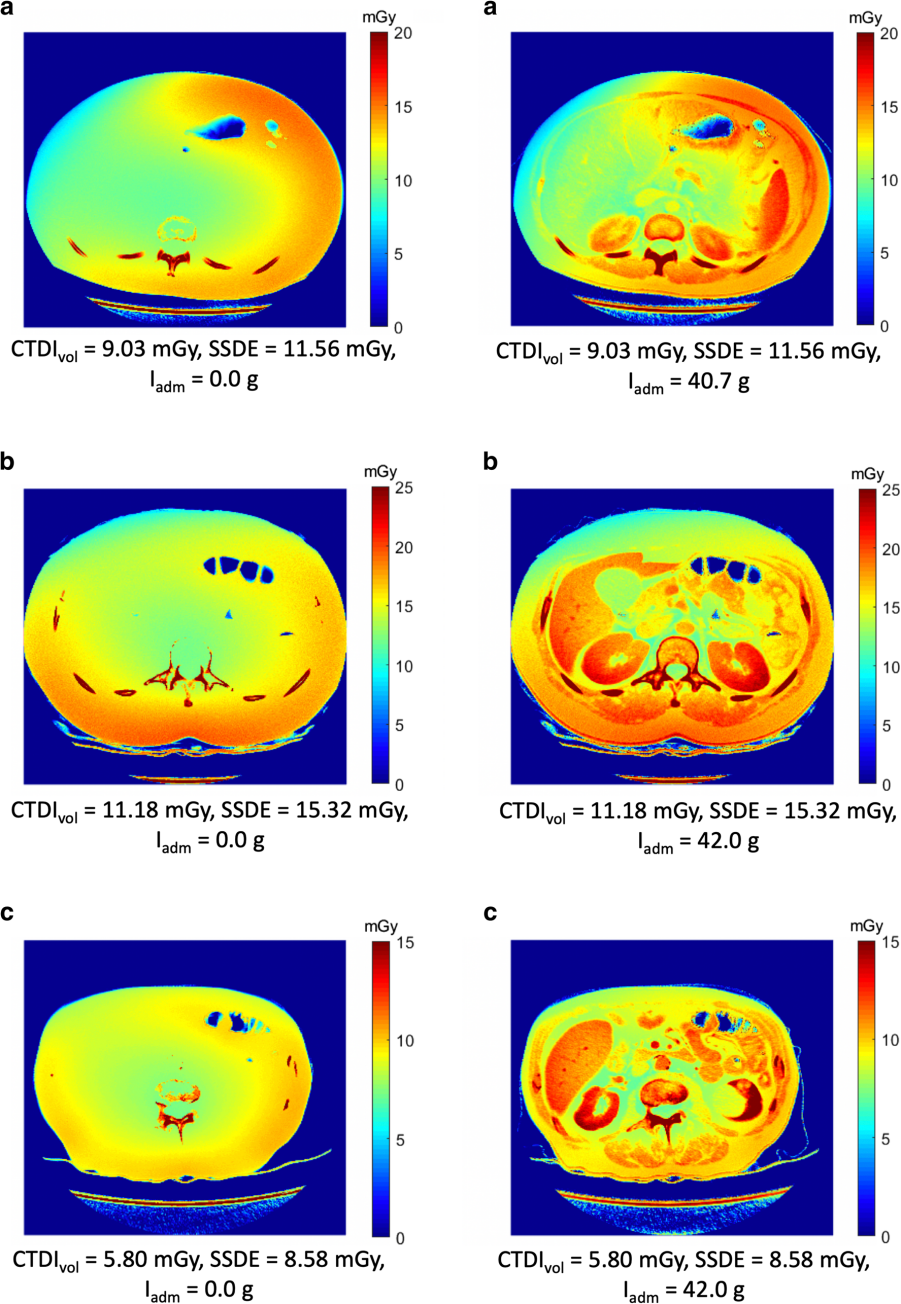
Parametric dose maps of three patients in the absence (left column) and presence (right column) of CA. **a** 23-year-old female administered to 370 mg I/ml (Iopromide), (**b**) 35-year-old female administered to 350 mg I/ml (Iomeprol), (**c**) 76-year-old male administered to 350 mg I/ml (Iomeprol), Abbreviations: I_adm_, administered iodine

Results of the patient study are shown in Figs. 3, 4, and 5 and Tables 1 and 2. For all the patients (PA), the D_SSDE_ increases in all the organs in the presence of CA (Fig. 3), and this increase is directly related to the iodine content in the organs (Table 1). The highest increase in the D_SSDE_ is seen in the left kidney (39%), followed by the right kidney (37%), aorta (34%), spleen (26%), liver (21%), and the lowest in the liver parenchyma (20%). The highest increase in HU values is seen in the left kidney (157HU), and the lowest is seen in the liver parenchyma (67HU) (Table 1). For each patient, the mean absolute organ doses (excluding aorta) in the presence $\left({\overline{\mathrm{D}}}_{\mathrm{I}}\right)$ and absence of contrast $\left({\overline{\mathrm{D}}}_0\right)$ and their ratio (DEF) are reported in Table 2, illustrating a maximum of 38% (PA7) and minimum of 21% (PA13) increase in the mean absolute organ doses. By applying a Pearson correlation test between the %Dose_SSDE_ increase and iodine content in all the organs, a linear increase is noticed (Fig. 4a, *R*^2^ = 0.86 and *p*_value_ < 0.01). In addition, the D_SSDE_ of patient study are in good agreement with the D_SSDE_ of the phantom study (Fig. 4b).

**Table 1:** The mean HU values ± SD, the mean iodine concentrations ± SD, and the D_SSDE_ ± SD in the segmented organs and tissues of the 20 patients

Parameter	Liver	Liver parenchyma	Right kidney	Left kidney	Aortic blood	Spleen
Mean CT values (HU) ± SD in VUE images	50 ± 12	49 ± 14	34 ± 5	34 ± 4	50 ± 5	44 ± 4
Mean CT values (HU) ± SD in CE images	120 ± 32	116 ± 33	190 ± 42	191 ± 39	131 ± 19	173 ± 30
Mean iodine concentration ± SD (mg I/ml)	3.00 ± 0.91	2.85 ± 0.86	6.38 ± 1.60	6.43 ± 1.50	5.32 ± 1.10	3.48 ± 0.71
Mean dose increase ± SD (mGy)	21 ± 5%	20 ± 5%	37 ± 7%	39 ± 7%	34 ± 6%	26 ± 4%

*Abbreviations: HU* Hounsfield units, *SD* standard deviation, *VUE* virtual-unenhanced, *CE* contrast-enhanced, *SSDE* size-specific dose estimate

**Table 2:** The administered iodine, CTDI_vol_, SSDE, mean absolute dose ± SD in the absence of contrast, mean absolute dose ± SD in the presence of contrast, and the dose enhancement factor ± SD (DEF) in the organs and tissues (excluding aorta) for each patient

Patient	Administered iodine (g)	CTDI_vol_ (mGy)	SSDE (mGy)	$\overline{D}$_0_ (mGy)	$\overline{D}$_I_ (mGy)	DEF =$\frac{\overline{D_I}}{\overline{D_0}}$
PA1	38.5	9.42	11.59	10.16 ± 0.42	12.70 ± 0.98	1.25 ± 0.11
PA2	42.0	10.10	12.42	10.88 ± 0.31	13.32 ± 0.92	1.22 ± 0.09
PA3	38.5	9.03	11.92	10.83 ± 0.59	13.53 ± 1.30	1.25 ± 0.14
PA4	42.0	11.18	15.32	13.84 ± 0.42	18.04 ± 1.56	1.30 ± 0.12
PA5	36.7	7.95	12.16	10.88 ± 0.65	13.64 ± 1.43	1.25 ± 0.15
PA6	42.0	9.03	11.92	10.44 ± 0.37	13.99 ± 0.50	1.34 ± 0.07
PA7	42.0	5.80	8.58	8.11 ± 0.26	11.21 ± 1.45	1.38 ± 0.18
PA8	44.4	9.03	12.37	9.74 ± 0.69	12.97 ± 1.39	1.33 ± 0.17
PA9	42.0	7.95	10.18	8.65 ± 0.64	10.78 ± 1.19	1.25 ± 0.17
PA10	42.0	10.10	12.42	11.55 ± 0.80	15.28 ± 2.09	1.32 ± 0.20
PA11	44.4	12.25	14.58	13.08 ± 0.43	17.43 ± 1.07	1.33 ± 0.09
PA12	42.0	7.95	11.77	11.45 ± 0.27	14.57 ± 1.25	1.27 ± 0.11
PA13	40.7	9.03	11.56	10.70 ± 0.53	12.92 ± 1.76	1.21 ± 0.17
PA14	42.0	7.93	9.04	7.25 ± 0.60	9.41 ± 1.02	1.30 ± 0.18
PA15	38.8	10.10	12.02	10.45 ± 0.81	13.04 ± 1.26	1.25 ± 0.15
PA16	42.0	7.95	11.37	9.96 ± 0.35	12.50 ± 1.07	1.26 ± 0.12
PA17	30.4	11.18	15.32	13.29 ± 1.51	16.62 ± 2.42	1.25 ± 0.23
PA18	44.4	9.03	11.92	11.13 ± 1.15	14.64 ± 1.70	1.32 ± 0.20
PA19	35.2	7.73	11.83	10.53 ± 0.67	14.28 ± 1.82	1.36 ± 0.19
PA20	42.0	12.25	11.27	9.40 ± 1.19	11.90 ± 1.43	1.27 ± 0.22

*Abbreviations: g* gram, *CTDI*_*vol*_ volume computed tomography dose index, *mGy* milli-gray, *SSDE* size-specific dose estimate, *SD* standard deviation*,* ${\overline{D}}_0$ mean absolute dose in the absence of contrast agent excluding aorta*,* ${\overline{D}}_I$ mean absolute dose in the presence of contrast agent excluding aorta, *DEF* dose enhancement factor

The impact of the patient’s anatomy and contrast distribution on radiation dose distribution is illustrated in Fig. 5. The parametric dose maps show three patients with a respectively low, moderate, and high increase in the absorbed radiation dose due to iodine contrast.

## Discussion

This study confirmed that the presence of CA increases the organ radiation dose in CT (Figs. 2 and 3). In the phantom study, a linear relation between the CTDI_vol_ normalized radiation dose and clinical range of CA concentrations was observed (Fig. 2a). In the patient study, data demonstrate that lower CA administrations result in lower doses (Fig. 4). The D_SSDE_ in the organs increased in the presence of CA, and this increase was also linear. The maximum and minimum increase in the dose was observed in the kidneys (37–39%) and liver parenchyma (20%) (Table 1). The high dose increase in the kidneys is caused by their high vascularization and high content of contrast at the moment of CT acquisition. The low dose increase in Fig. 4 a belongs to a patient who is diagnosed with ascites. The accumulated fluid attenuates the beam, resulting in a lower dose in the organs. Overall, the interpatient variability in the organ dose values can be attributed to the patient’s anatomy, physiology, location of the organ in the body, and iodine administration. The patient-dependent variability in the results (Figs. 4b and 5) motivates patient-specific dosimetry approaches for more accurate dose estimation in CT.

The knowledge of CA distribution in tissues is a prerequisite for dosimetry calculations following contrast-enhanced CT. Different strategies have been deployed to estimate the amount of CA in the organs. Amato et al obtained the HU-iodine density relation with mathematical equations, and used a simplified HU-based geometrical model. They reported up to a 74% dose increase [13]. Sahbaee et al used a pharmacokinetic human model and reported up to a 54% dose increase [14]. Perisinakis et al performed experimental measurements to obtain HU-iodine density relation. They used mathematical anthropomorphic phantoms and reported up to a 100% dose increase [15]. Comparing between the liver, kidneys, aorta, and spleen, these studies reported the maximum organ dose in kidneys [13–15], which is comparable to our results. In this study, we used the iodine-HU calibration curve of the CT scanner for the phantom study and the iodine quantification of DECT in voxelated patient-specific CT models. We expect a more precise estimation with our approach: (i) iodine quantification in DECT is reported to be accurate (± 10 % ,[30]), (ii) DECT can be used to precisely estimate the iodine content of organs in each image voxel at the time of scan and take into account the tissue heterogeneity, and (iii) DECT is offering the opportunity to investigate the net effect of CA by providing a same patient geometry and exposure parameters in the presence and absence of CA. An alternative for DECT could be the use of unenhanced and contrast-enhanced images of conventional CT scans. However, the absence of iodine quantifications and the differences between exposures make the radiation dose comparison increasingly difficult. The clinical relevance of absorbed radiation dose on biological damage is not yet fully known; nonetheless, a different extent of biological damage is expected in different types of tissue. The impact of iodine on biological radiation damage is assessed by investigating the increase in the DNA DSB [5–10]. However, not all the DNA DSB are malignant transformation due to fast regeneration of blood cells. The biological damage of parenchymal tissue occurs if iodine reaches the capillary network of an organ or the interstitial fluid outside these vessels [33]. A recent study showed that the secondary electrons can escape vessel walls that are smaller than 100 μm and cause biological damage to the surrounding tissue [2]. Consequently, the increase of absorbed dose in organs that contain parenchymal tissue such as the liver, kidneys, lungs, and brain may lead to biological damage and should be considered. We excluded the absorbed dose of aortic blood for reporting the average of the absolute dose values in Table 2 in order to make the results clinically more relevant.

A limitation of this study was using a single type of CT scanner. However, we expect that the normalization of the data to SSDE provides results that are scanner and patient size independent. Another limitation was performing the simulations only for abdominal scans. For other CT exams, different results might be expected depending on the type and moment of CT acquisition and the iodine administration. The other limitation is the presence of three brands of CA in the patient study. However, we believe that if the iodine concentration (mg I/mL) of the CA is respected, that is very unlikely that the brand or type would change the result considering the strict relationship between iodine concentration and CT attenuation. Another limitation of this study is using VUE images instead of true unenhanced images. From a dosimetric point of view, we believe that the use of VUE images is acceptable and it would not have an important impact on the results compared to true unenhanced images, as the HU values are similar between both [34, 35]. Also, since this study was limited to the retrospective data obtained from hepatic-phase abdominal scans, we did not investigate the impact of different scan phases on the organ absorbed doses. Further research should be undertaken to explore the impact of scan phase on absorbed organ doses.

In summary, this study showed that using CA in CT leads to an average organ dose increase of 30%. Despite the extensive use of CA in clinic, most of dosimetry studies do not account for the impact of CA on radiation dose. Considering the role of CA on increasing the absorbed radiation dose and increasing the X-ray-induced DNA DSB [5–10], it is reasonable to consider the presence of CA in dosimetry calculations and epidemiological studies which investigate the impact of radiation on health effects. Finally, considering that contrast-enhanced CT is necessary for many diagnostic procedures, this study suggests including the effect of contrast agent on organ dose while developing new CT protocols.

## Abbreviations

${\overline{\mathrm{D}}}_0$: Mean absolute dose in the absence of contrast agent
${\overline{\mathrm{D}}}_{\mathrm{I}}$: Mean absolute dose in the presence of contrast agent
CA: Contrast agent
CTDI: Computed tomography dose index
D: Dose
DECT: Dual-energy computed tomography
DEF: Dose enhancement factor
DLP: Dose length product
DSB: Double-strand breaks
D_SSDE_: Mean dose normalized by size-specific dose estimate
E: Effective dose
I_adm_: Administered iodine
I_conc_: Iodine concentration
MC: Monte Carlo
PA: Patient
PMMA: Polymethyl methacrylate
SSDE: Size-specific dose estimate
V_I_: Dose volume in the presence of contrast agent
V_I0_: Dose volume in the absence of contrast agent
VUE: Virtual-unenhanced

## Declarations

### Guarantor

The scientific guarantor of this publication is Prof. Nico Buls.

### Statistics and biometry

One of the authors has significant statistical expertise.

### Methodology

• retrospective

• experimental/observational

• performed at one institution

## References

[1] Sahbaee P, Segars WP, Marin D (2017). The effect of contrast material on radiation dose at CT: part I. Incorporation of Contrast Material Dynamics in Anthropomorphic Phantoms. Radiology.

[2] Harbron RW, Ainsbury EA, Bouffler SD (2018). The impact of iodinated contrast media on intravascular and extravascular absorbed doses in X-ray imaging: a microdosimetric analysis. Phys Med.

[3] American College of Radiology (2020). ACR manual on contrast media.

[4] Van der Molen AJ, Reimer P, Dekkers IA (2018). Post-contrast acute kidney injury. Part 2: risk stratification, role of hydration and other prophylactic measures, patients taking metformin and chronic dialysis patients: Recommendations for updated ESUR Contrast Medium Safety Committee guidelines. Eur Radiol.

[5] Grudzenski S, Kuefner MA, Heckmann MB, Uder M, Lobrich M (2009). Contrast medium-enhanced radiation damage caused by CT examinations. Radiology.

[6] Piechowiak EI, Peter J-FW, Kleb B, Klose KJ, Heverhagen JT (2015). Intravenous iodinated contrast agents amplify DNA radiation damage at CT. Radiology.

[7] Van Cauteren T, Honoria Da Silva E, Van Gompel G (2019). Iodine dose of administered contrast media affects the level of radiation-induced DNA damage during cardiac CT scans. AJR Am J Roentgenol.

[8] Deinzer CKW, Danova D, Kleb B, Klose KJ, Herverhagen JT (2014). Influence of different iodinated contrast media on the induction of DNA double-strand breaks after in vitro X-ray irradiation. Contrast Media Mol Imaging.

[9] Wang L, Li Q, Wang X-M (2017). Enhanced radiation damage caused by iodinated contrast agents during CT examination. Eur J Radiol.

[10] Gould R, McFadden SL, Horn S, Prise KM, Doyle P, Hughes CM (2016). Assessment of DNA double-strand breaks induced by intravascular iodinated contrast media following in vitro irradiation and in vivo, during paediatric cardiac catheterization. Contrast Media Mol Imaging.

[11] Marin D, Nelson RC, Rubin GD, Schindera ST (2011). Body CT: technical advances for improving safety. AJR Am J Roentgenol.

[12] Huda W, Scalzetti EM, Levin G (2000). Technique factors and image quality as functions of patient weight at abdominal CT. Radiology.

[13] Amato E, Lizio D, Settineri N, Di Pasquale A, Salamone I, Pandolfo I (2010). A method to evaluate the dose increase in CT with iodinated contrast medium. Med Phys.

[14] Sahbaee P, Abadi E, Segars WP, Marin D, Nelson RC, Samei E (2017). The effect of contrast material on radiation dose at CT: part II. A Systematic Evaluation across 58 Patient Models. Radiology.

[15] Perisinakis K, Tzedakis A, Spanakis K, Papadakis AE, Hatzidakis A, Damilakis J (2018). The effect of iodine uptake on radiation dose absorbed by patient tissues in contrast enhanced CT imaging: implications for CT dosimetry. Eur Radiol.

[16] The American Association of Physicists in Medicine (2008), AAPM Reports - the measurement, reporting, and management of radiation dose in CT. The American Association of Physicists in Medicine, Alexandria. Available via https://www.aapm.org/pubs/reports/detail.asp?docid=97. Accessed 14 Jan 2020

[17] Stamm G, Nagel H (2003). CT-Expo - a novel program for dose evaluation in CT. Rofo.

[18] Lee C, Kim KP, Bolch WE, Moroz BE, Folio L (2015). NCICT: a computational solution to estimate organ doses for pediatric and adult patients undergoing CT scans. J Radiol Prot.

[19] Deak P, van Straten M, Shrimpton PC, Zankl M, Kalender WA (2008). Validation of a Monte Carlo tool for patient-specific dose simulations in multi-slice computed tomography. Eur Radiol.

[20] Chen W, Kolditz D, Beister M, Bohle R, Kalender WA (2012). Fast on-site Monte Carlo tool for dose calculations in CT applications. Med Phys.

[21] Hupfer M, Kolditz D, Nowak T, Eisa F, Brauweiler R, Kalender WA (2012). Dosimetry concepts for scanner quality assurance and tissue dose assessment in micro-CT. Med Phys.

[22] Kelaranta A, Mäkelä T, Kaasalainen T, Kortesniemi M (2017). Fetal radiation dose in three common CT examinations during pregnancy – Monte Carlo study. Phys Med.

[23] Myronakis M, Perisinakis K, Tzedakis A, Gourtsoyianni S, Damilakis J (2009). Evaluation of a patient-specific Monte Carlo software for CT dosimetry. Radiat Prot Dosim.

[24] Damilakis J, Perisinakis K, Tzedakis A, Papadakis A, Karantanas A (2010). Radiation dose to the conceptus from multidetector CT during early gestation: a method that allows for variations in maternal body size and conceptus position. Radiology.

[25] Nuclear Energy Agency (2018). NEA - PENELOPE 2018: a code system for Monte Carlo simulation of electron and photon transport: Workshop Proceedings.

[26] Allison J, Amako K, Apostolakis J (2016). Recent developments in Geant4. Nucl Instrum Methods Phys Res Sect A.

[27] Werner CJ, Bull JS, Solomon CJJ et al (2018) MCNP6.2 Release Notes, A General Monte Carlo N-Particle (MCNP) Transport Code, report LA-UR-18-20808. Los Alamos National Laboratory, USA. Available via https://mcnp.lanl.gov/pdf_files/la-ur-18-20808.pdf. Accessed 25 Aug 2020

[28] Nelson WR, Hirayama H, Rogers DWO (1985). EGS4 code system.

[29] Allison MA, Kwan K, DiTomasso D, Wright CM, Criqui MH (2008). The epidemiology of abdominal aortic diameter. J Vasc Surg.

[30] Jacobsen MC, Schellingerhout D, Wood CA (2018). Intermanufacturer comparison of dual-energy CT iodine quantification and monochromatic attenuation: a phantom study. Radiology.

[31] Fedorov A, Beichel R, Kalpathy-Cramer J (2012). 3D Slicer as an image computing platform for the Quantitative Imaging Network. Magn Reson Imaging.

[32] The American Association of Physicists in Medicine (2011) AAPM Reports - size-specific dose estimates (SSDE) in pediatric and adult body CT examinations. The American Association of Physicists in Medicine, Alexandria. Available via https://www.aapm.org/pubs/reports/detail.asp?docid=143. Accessed 3 Jul 2019

[33] Harbron R, Ainsbury EA, Bouffler SD, Tanner RJ, Eakins JS, Pearce MSL (2017). Enhanced radiation dose and DNA damage associated with iodinated contrast media in diagnostic X-ray imaging. Br J Radiol.

[34] Li Y, Li Y, Jackson A (2017). Comparison of virtual unenhanced CT images of the abdomen under different iodine flow rates. Abdom Radiol (NY).

[35] Graser A, Johnson TRC, Hecht EM (2009). Dual-energy CT in patients suspected of having renal masses: can virtual nonenhanced images replace true nonenhanced images?. Radiology.

